# Flaxseed Improves Glucose and Lipid Metabolism in Mexican Subjects with Type 2 Diabetes: A Parallel Randomized Clinical Trial

**DOI:** 10.3390/nu17040709

**Published:** 2025-02-17

**Authors:** Sabina López-Toledo, María Cruz Pineda De la Cruz, Itzae Adonai Gutiérrez-Hurtado, Ana L. Gijón-Soriano, Enrique Martínez-Martínez, Carlos Valencia-Santiago, José E. Orellana-Centeno, Sergio A. Ramírez-García, Royer Pacheco-Cruz

**Affiliations:** 1 Center for Studies in Health Sciences and Disease, “Benito Juarez” Autonomous University of Oaxaca, Oaxaca 68000, Mexico; 2 “Presidente Benito Juárez” Hospital, Oaxaca 68040, Mexico; maris90_7@hotmail.com; 3 Department of Molecular Biology and Genomics, University Center for Health Sciences, University of Guadalajara, Guadalajara 44340, Mexico; itzae.gutierrez@academicos.udg.mx; 4 Faculty of Dentistry, “Benito Juarez” Autonomous University of Oaxaca, Oaxaca 68120, Mexico; analigiso2807@gmail.com (A.L.G.-S.); quique1263@hotmail.com (E.M.-M.); orellana17@msn.com (J.E.O.-C.); 5 Faculty of Medicine, “Benito Juarez” Autonomous University of Oaxaca, Oaxaca 68000, Mexico; l.n.carlosvalencia23@gmail.com; 6 Faculty of Chemical Sciences, “Benito Juarez” Autonomous University of Oaxaca, Oaxaca 68000, Mexico; sergionabmsp@gmail.com; 7 Health and Civil Protection Directorate of Ejutla de Crespo, Oaxaca 71500, Mexico; roger_0912@hotmail.es

**Keywords:** parallel randomized clinical trial, flaxseed, type 2 diabetes, glucose metabolism, lipid metabolism

## Abstract

Introduction: Type 2 diabetes is a significant health concern in the 21st century, and its prevalence continues to rise despite efforts to promote preventive lifestyle changes. This increase has led to higher economic burdens, prompting the search for non-pharmacological methods to manage glucose levels. Objective: To assess the effects of flaxseed consumption on biochemical markers (glucose, glycated hemoglobin, total cholesterol, and triglycerides) in adult patients with Type 2 diabetes in Oaxaca, Mexico. Materials and Methods: Participants were recruited and randomized into clinical trials between April and June 2023, and the study protocol was approved by a Human Research Ethics Committee. Results: Consuming 16 g of flaxseed daily for three months led to a significant decrease in glucose, total cholesterol, and triglyceride levels (*p* < 0.001) in Type 2 diabetes patients in the intervention group (*n* = 82). Belonging to the control group (*n* = 84) was correlated with presenting higher levels of glycated hemoglobin (Spearman’s Rho 0.640; *p* < 0.001), higher levels of glucose (Spearman’s Rho 0.352; *p* < 0.001), total cholesterol (Spearman’s Rho 0.796; *p* < 0.001), and triglycerides (Spearman’s Rho 0.700; *p* < 0.001). Conclusions: A daily intake of 16 g of flaxseed is an effective supplementary treatment for adult Mexican patients with Type 2 diabetes, as evidenced by reduced levels of glycated hemoglobin, glucose, cholesterol, and triglycerides in the intervention group. Potential implications for clinical practice: Healthcare providers may consider recommending flaxseed in the diets of patients with obesity, prediabetes, or Type 2 diabetes to improve glucose and lipid metabolism and overall metabolic health.

## 1. Introduction

Type 2 diabetes is one of the most important health emergencies of the 21st century. It is estimated that there are 463 million people worldwide with Type 2 diabetes, and that this number may increase to 578 million by 2030 and 700 million by 2045. Hyperglycemia is associated with acute and chronic complications, and in both cases, it affects the cardiovascular system, thereby inherently affecting the entire organism, but mainly the eyes, kidneys, and peripheral nerves [1,2].

Despite the health authorities’ insistence on the population making changes to their lifestyle and hygienic-dietary habits to prevent this disease, the number of patients with Type 2 diabetes continues to grow exponentially, which leads to an increase in the country’s economic expenditure. Non-pharmacological measures to reduce glycemia in these patients are continuously sought. In this sense, the consumption of functional foods represents a very viable option, due to their nutritional, economic, and practical characteristics [3,4].

Different studies in Mexico have shown that, within functional foods, the use of seeds is an excellent option for various reasons. In Mexico, the use of seeds for different culinary preparations is common; even in Oaxaca, the second-lowest socioeconomic state, there are programs where strategies have been implemented that use these seeds to promote healthy eating. One of the seeds that presents the most scientific evidence is flaxseed, demonstrating its positive effect on the reduction in biochemical levels of cholesterol, triglycerides, and glucose, due to its high fiber content [5,6,7,8,9].

The lignans present in flaxseed, due to their antioxidant properties, help reduce the risk of oxidative damage to DNA (deoxyribonucleic acid) [10], reduce lipid peroxidation, and prevent oxidative stress [11], promoting the production of polyunsaturated fatty acids EPA (eicosapentaenoic acid) and DHA (docosahexaenoic acid) [12,13]. Furthermore, the fiber contained in flaxseed is 25% insoluble and 75% soluble (gums, pectin, and glutamate), so it plays an important role in reducing plasma glucose, during the absorption of cholesterol and triglycerides, and is important for the metabolism of the microbiota [14]. Similarly, it reduces inflammation [15] through its influence on eicosanoids, crucial elements for the prevention of Type 2 diabetes [16,17,18,19,20,21,22]. A recent study confirmed its efficacy in improving hepatic lipid deposition, liver function, body composition indicators, and lipid metabolism in patients with non-alcoholic liver disease, in whom it also regulated the intestinal microbiota, increasing the abundance of beneficial bacteria and reducing harmful bacteria [23].

The primary objective of this study was to evaluate the impact of flaxseed consumption on key biochemical markers—glucose, glycated hemoglobin, total cholesterol, and triglycerides—in adult patients with Type 2 diabetes from Oaxaca, Mexico. Secondary objectives included the following: (1) assessing and describing the clinical characteristics of these patients, including family history of hypertension and Type 2 diabetes, smoking habits, and drug use; (2) providing 16 g of flaxseed daily for three months to the intervention group; and (3) measuring and describing the biochemical parameters (glucose, glycated hemoglobin, total cholesterol, and triglycerides) of patients with Type 2 diabetes at baseline and following the flaxseed intervention.

## 2. Materials and Methods

### 2.1. Participants

Participants were recruited from the Nutrition outpatient clinic of the Oaxaca “Presidente Benito Juárez” Hospital. Ther inclusion criteria included the following: being eligible patients from the Nutrition outpatient clinic of the “Presidente Benito Juárez” Hospital, Oaxaca, Mexico; patients with a diagnosis of uncontrolled Type 2 diabetes evidenced by biochemical markers valid for a maximum of 1 month (fasting blood glucose greater than 140 and glycated hemoglobin greater than 6.5%); patients with regular attendance at the outpatient clinic; minimum attendance of 3 consecutive months; and an indication for metformin therapy.

Exclusion criteria included patients with chronic kidney disease, patients receiving renal replacement therapy, perimenopausal or menopausal women, patients with glucose disorders secondary to autoimmune Type 2 diabetes, patients with thyroid disease or insulin-producing tumors, patients with a history of poor medication adherence, patients with literacy challenges, patients without reliable means of contact for follow-up, and patients with gestational diabetes. The sample calculation was as follows: accepting an alpha risk of 0.05 and a statistical power of >0.8 in a two-tailed test, 79 cases and 79 controls are required. It was assumed that the rate of exposure in the control group would be 0.4. A loss to follow-up rate of 15% was estimated.

### 2.2. Aims and Research Questions

The primary objective of the study was to assess the effects of flaxseed consumption on biochemical markers (glucose, glycated hemoglobin, total cholesterol, and triglycerides) in adult patients with Type 2 diabetes from the state of Oaxaca, Mexico. The secondary objectives were as follows: to assess and describe the clinical characteristics (family history of hypertension and Type 2 diabetes, smoking, or drug addiction) of patients with Type 2 diabetes; to provide 16 g of flaxseed per day for three months to the members of the intervention group; and to assess and describe the biochemical parameters (glucose, glycated hemoglobin, total cholesterol, and triglycerides) of patients with Type 2 diabetes (baseline and after flaxseed intervention).

The research question was as follows: Does adjuvant treatment with metformin plus 16 g of flaxseed per day for three months have a beneficial effect on biochemical markers (glucose, glycated hemoglobin, total cholesterol, and triglycerides) in adult patients with uncontrolled Type 2 diabetes from Oaxaca, Mexico?

### 2.3. Study Design

This was a randomized, parallel-group clinical trial with a 1:1 allocation ratio. Participants were recruited, screened, and randomized for this clinical trial between April and June 2023. The clinical trial was registered (ClinicalTrials.gov identification: NCT06683235) and the study protocol was approved by the Human Research Ethics Committee of the Universidad de la Sierra Sur (registration number: CEI-04-2023, approval date: 20 April 2023), according to the Declaration of Helsinki’s statement. All participants were informed about the study and signed written informed consent.

The primary outcome was the reduction in HbA1c at 12 weeks. Secondary outcomes included fasting blood glucose and lipid profile changes. This was an open-label study, meaning that both participants and researchers were aware of the assigned intervention.

The randomization sequence was computer-generated with a permuted block design, (block size = 88), with a 1:1 allocation ratio. For allocation concealment, sequentially numbered opaque sealed envelopes were employed, preventing the assignment from being revealed until the time of allocation.

An independent statistician generated the random sequence using a computerized randomization program in R. A researcher not involved in data collection or analysis performed patient enrollment and allocation to intervention groups.

Patients that signed the informed consent were randomly allocated into a group, with those with an even number in the research file assigned to the control group (A) and those with an odd number assigned to the intervention group (B).

No modifications were made to the study protocol, eligibility criteria, or outcome measures after the trial commenced.

Group A (control group). They were given verbal instructions to continue with the treatment established by the treating physician, given basic nutritional guidance with the help of the healthy eating plate, and informed that during the study, they could not consume alcoholic beverages or supplements during the three months of the intervention.

Group B (intervention group). They were given verbal instructions to continue with the treatment established by the treating physician, received basic nutritional guidance with the help of the healthy eating plate, and were given 1440 g of flaxseed (16 g daily for 90 days), divided into 8 g bags to consume one 8 g spoonful of flaxseed at 10 a.m., and 8 g of flaxseed at 5 p.m., for 3 months. They were informed that they could not consume alcoholic beverages during the study, and they were given a brochure with photos about the different possible ways of consuming flaxseed and its benefits. The WhatsApp numbers of the participants were saved to maintain frequent contact, remind them to take the flaxseed, and ask about possible symptoms or unforeseen events.

### 2.4. Screening

At the screening visit, participants were asked to complete a screening questionnaire about their age, gender, diabetes diagnosis, current medications, and clinical characteristics (family history of diabetes, hypertension, and dyslipidemia, presence of alcohol, smoking, and drug addiction).

Next, participants were instructed to attend the clinical laboratory on an empty stomach between 7 and 10 a.m., where the sample was collected and analyzed by the hospital laboratory manager, and the procedure was repeated 12 weeks later.

Glucose was measured by using the glucose oxidase method (ADVIA, 208 model 2400, Siemens Healthcare Diagnostics S.A.; Erlangen, Germany) and glycated hemoglobin (HbA1c) was determined via turbidimetric inhibition immunoassay (COBAS, model C 513, Roche Diagnostica LTDA., Toluca, Mexico), with a sensitivity of <4.2% and a within-run CV of 1.6%. Total cholesterol and triglycerides were measured using the cholesterol esterase/cholesterol oxidase/peroxidase method and the lipase/glycerol kinase/glycerol-3-phosphate oxidase/peroxidase method, respectively. All biochemical measurements were performed using standardized protocols and were subject to internal quality control. Calibration was performed using certified reference materials.

The results were compared with the normal figures for this age group, according to the current regulations: Glucose (normal 70–99 mg/dL; prediabetes 100–125 mg/dL; diabetes >126 mg/dL). Glycated hemoglobin (normal <5.6%; prediabetes 5.7–6.4%; diabetes >6.5%). Total cholesterol (normal <200 mg/dL), triglycerides (normal <150 mg/dL)

### 2.5. Experimental Protocol

The researchers purchased, packaged, and provided the flaxseed powder to the members of group B. The flaxseed was delivered in packets containing 8 g of ground flaxseed each, which were given to the patients for daily consumption (one 8 g spoonful of flaxseed in the morning at 10 a.m. and one 8 g spoonful of flaxseed in the afternoon at 5:00 p.m.). Leaflets with images explaining how to consume it and how the glass should look after finishing were provided. Various ways of consuming the powder were also provided to avoid the subjects getting bored with the taste and consistency of the flaxseed. According to previous studies, it has been observed that the administration of between 15 and 30 g of flaxseed improves glycemic control and lipid profiles, this indicates that a dose of 15 g of flaxseed is sufficient to inhibit the alpha-glucosidase and alpha-amylase enzymes [24,25]. For this reason, it was decided to administer 16 g of flaxseed, both based on the evidence and to comply with the WHO and ADA recommendation of 25 g to 30 g of fiber per day, considering that there would be an extra fiber contribution from vegetables and fruits consumed in the diet.

### 2.6. Monitoring and Surveillance

Daily contact was maintained with the participants via WhatsApp in order to remind them to take the flaxseed and also to find out if they had any adverse symptoms (abdominal distension, flatulence, gas, or diarrhea, etc.), as well as to resolve doubts and ask if they were taking other vitamins or medications.

Participants lost to follow-up were documented, and their reasons for withdrawal were recorded. A total of 10 participants (6 from the intervention group and 4 from the control group) were lost to follow-up, primarily due to non-adherence to the intervention.

### 2.7. Statistical Analysis

Statistical analysis was performed using IBM SPSS Statistics version 23. Descriptive statistics, including means, standard deviations, and frequencies, were calculated. Group comparisons were performed using *t*-tests for continuous variables and chi-square tests for categorical variables, depending on the nature of the data. For within-group comparisons (baseline vs. 3-month follow-up), paired *t*-tests were applied. Pearson’s correlation coefficient was used to examine the relationships between continuous variables. Assumptions of normality and homogeneity of variances were tested (Kolmogorov–Smirnov), and non-parametric tests were applied when necessary. A value of *p* < 0.05 was considered statistically significant.

## 3. Results

**Figure 1:**
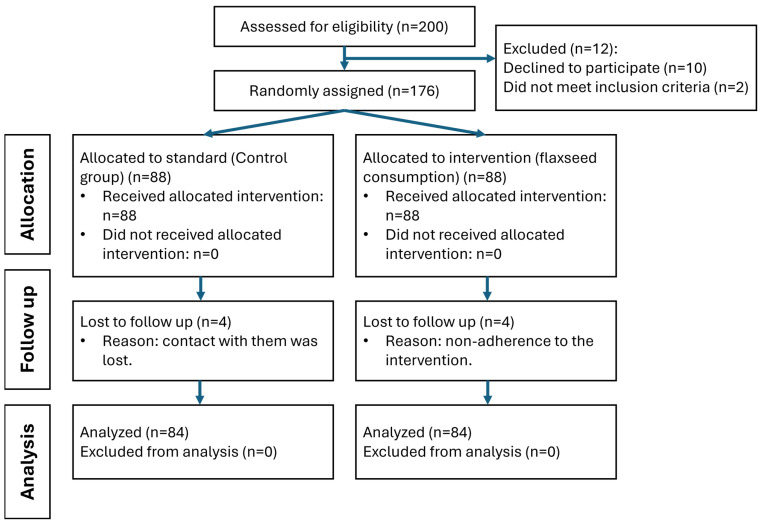
Study participants’ flowchart (*n* = 166).

The screening visit was attended by 200 subjects, and 166 participants (84 in the control group and 82 in the intervention group) completed the full study protocol (Figure 1).

### 3.1. Baseline Characteristics of Study Participants

Table 1 shows the sociodemographic, clinical, and biochemical characteristics of the participants in the control group (*n* = 84) and intervention group (*n* = 82) before the intervention.

### 3.2. Behavior of Biochemical Parameters

**Figure 2:**
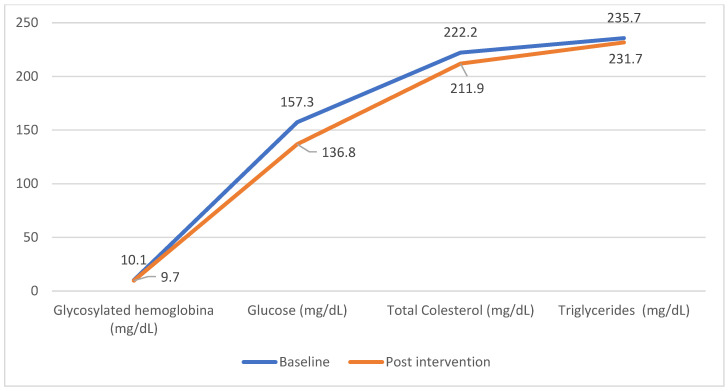
Behavior of biochemical parameters of participants in the control group.

Figure 2 shows the behavior of the biochemical parameters of the control group. Although a decrease was observed, the differences are not significant (W of Wilcoxon).

**Figure 3:**
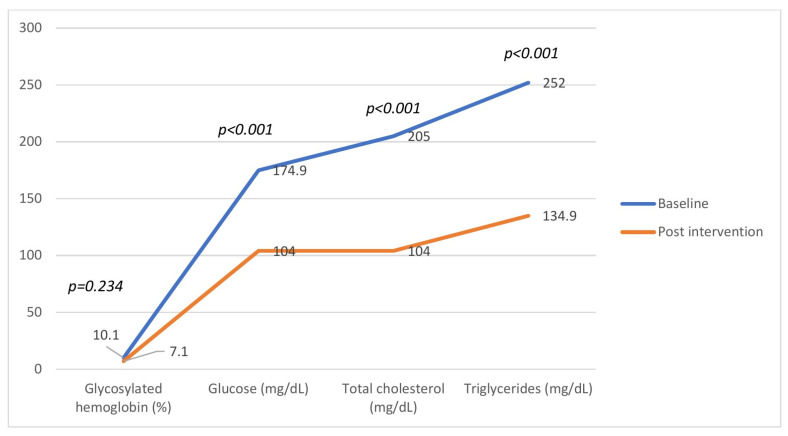
Behavior of biochemical parameters of participants in the intervention group (*n* = 82) before and after 12 weeks of flaxseed supplementation. Significant reductions were observed in fasting glucose, total cholesterol, and triglycerides (*p* < 0.001), while the change in glycated hemoglobin was not significant (*p* = 0.234). Data are presented as mean ± SD.

Figure 3 shows the behavior of the biochemical parameters of the intervention group. A significant decrease in glucose, total cholesterol, and triglyceride levels was observed (W of Wilcoxon: *p* < 0.001; *p* < 0.001; and *p* < 0.001).

Pearson correlation analysis was performed with the variables analyzed in this study, finding the following relations:

Belonging to the control group was correlated with presenting higher levels of glycated hemoglobin (Spearman’s Rho 0.640; *p* < 0.001), higher levels of glucose (Spearman’s Rho 0.352; *p* < 0.001), total cholesterol (Spearman’s Rho 0.796; *p* < 0.001), and triglycerides (Spearman’s Rho 0.700; *p* < 0.001).

The higher the total cholesterol levels at baseline, the higher the glycated hemoglobin (Spearman’s Rho 0.211; *p* = 0.006) and the higher the triglyceride levels (Pearson 0.223; *p* = 0.004) after the intervention. The higher the glucose levels at baseline, the higher the glucose levels (Spearman’s Rho 0.535; *p* < 0.001) after the intervention. The baseline level of glycated hemoglobin was positively correlated with the final glycated hemoglobin levels (Pearson 0.525; *p* < 0.001).

## 4. Discussion

The findings of this study demonstrate the efficacy of a 16 g/day flaxseed regimen as an adjuvant therapy for adult Mexican patients with Type 2 diabetes. Specifically, participants in the intervention group experienced a reduction in glycated hemoglobin, glucose, cholesterol, and triglyceride levels.

Evidence from various studies around the world suggests that flaxseed positively impacts glucose metabolism. Specifically, a 2022 systematic review and meta-analysis of randomized clinical trials [26] found that flaxseed supplementation improves glycemic control and insulin resistance in prediabetes and Type 2 diabetes.

A systematic review in 2018 [27], encompassing 25 randomized clinical trials, identified a significant association between flaxseed supplementation and a reduction in blood glucose. This effect was particularly evident with the administration of flaxseed powder at dosages ranging from 4 to 14 g [27]. The relationship was most significant in trials utilizing powdered flaxseed within the aforementioned dosage range. Consequently, the present study employs powdered flaxseed for supplementation. In September 2022, Moreira et al. [24] conducted a study in which men with Type 2 diabetes, aged 30 to 60 years, received 15 g of flaxseed on an empty stomach. The results indicated a decrease in postprandial glycemic response compared to the control group.

A 2017 randomized, cross-sectional study investigated the effects of flaxseed on blood glucose in individuals aged 35–45 [25]. Participants were divided into three groups: a control group consuming a 1350 kcal diet without flaxseed, a group consuming the same diet with 13 g/day of flaxseed oil six days per week, and a group consuming the base diet supplemented with 32 g/day of flaxseed flour. While all groups demonstrated a reduction in blood glucose levels, the flaxseed flour group exhibited the most significant improvement in fasting glucose [25]. In 2019, Hasaniani et al. [28] conducted a randomized controlled clinical trial to assess the impact of flaxseed on glycemic control in 57 patients with Type 2 diabetes. One group received 200 g/day of unflavored yogurt, while the intervention group received 200 g/day of unflavored yogurt with 30 g of flaxseed for a period of 8 weeks. The intervention group demonstrated a statistically significant reduction in glycated hemoglobin (*p* = 0.001) compared to the control group. These results strongly support those of this study, which demonstrated a significant reduction in glycated hemoglobin and glucose (*p* < 0.001) in participants receiving the flaxseed intervention.

Flaxseed doses between 14 and 30 g daily have yielded positive results in prior research. However, higher doses have also been associated with decreased participant adherence. Therefore, we selected a dose of 16 g of flaxseed per day for this study, balancing efficacy with tolerability. This dosage proved sufficient to observe promising improvements in biochemical markers, consistent with the positive outcomes seen with doses as low as 14 g in earlier studies [22,26].

The effects of flaxseed consumption on the lipid profile have also been previously studied. Morshedzadeh et al. [29] assessed the effectiveness of flaxseed supplementation for managing metabolic syndrome parameters, including serum concentrations of triglycerides, total cholesterol, and HDL/LDL, in patients with mild-to-moderate ulcerative colitis. The study included 70 participants who were randomly assigned to either an intervention group receiving 30 g of ground flaxseed daily for 12 weeks or a control group. The serum lipid and lipoprotein levels were evaluated at the beginning and end of the 12-week intervention period. The authors observed a significant reduction in serum concentrations of triglycerides and total cholesterol, as well as a significant increase in serum HDL levels, in the group receiving ground flaxseed powder. Flaxseed supplementation may aid in the treatment of dyslipidemia, particularly in overweight or obese patients. Moreover, the form of flaxseed consumed significantly impacts serum lipid and lipoprotein concentrations. Whole flaxseed is more effective in regulating lipid metabolism than flaxseed oil [30,31]. In a 2024 systematic review and meta-analysis, Ahmed et al. [32] observed that flaxseed supplementation offers beneficial modulation of blood lipid profiles (triglycerides, total cholesterol, HDL, and LDL) and liver enzymes. According to the literature, the beneficial effects of flaxseed supplementation may be due to its high soluble fiber content, which has a beneficial effect on the intestinal microbiota, which is essential in improving metabolic functions for the digestion and absorption of lipids, glucose, and the maintenance of the immune response [19]. Recent research by Tian et al. [23] supports these findings, demonstrating that the daily consumption of 30 g of flaxseed powder significantly improved gut microbiota diversity, as indicated by increases in the Shannon and Simpson indices. This enhancement led to improved glucose and lipid metabolism.

Research has shown that flax lignans can help prevent obesity or aid in weight loss by regulating adiponectin levels and up-regulating the level of fat oxidation in skeletal muscles. In addition, flax lignans could reduce the synthesis levels of the sterol regulatory elements necessary for TG production and increase short-chain fatty acid concentrations in the intestines, thereby enhancing satiety to promote weight loss [33]. Our findings align with previous studies, showing that 16 g of flaxseed daily for three months improves glycemic and lipid profiles in patients with uncontrolled Type 2 diabetes.

Flaxseed not only enhances biochemical health, but is also more cost-effective to consume directly than to purchase processed “natural products” containing flaxseed. In Oaxaca, Mexico, various flaxseed supplements are available, with the LIFE brand being one of the most popular among consumers. This brand offers capsules that include mallow, flaxseed markers, and aloe vera, priced at approximately $16.50. Another market option is the NOW brand flaxseed oil capsules, with a bottle of 180 capsules costing around $22.60. The most favored product in Oaxaca is SIMIFIBRA Forte, which consists of flaxseed powder; a 300 g bottle is priced at $14.60. In contrast, purchasing 500 g of flaxseed powder from traditional markets in Oaxaca City or throughout the state costs only $3.90, making it the most economically advantageous choice.

### 4.1. Perspectives for Clinical Practice

As the first study in Mexico to demonstrate the effectiveness of flaxseed in managing glucose and lipid metabolism in patients with Type 2 diabetes, this research has several potential implications for clinical practice.

Health system: The global economic burden of diabetes is considerable, with annual costs estimated at $370 billion, projected to increase to $490 billion by 2030 [34]. In Mexico, the direct medical expenses are significant, amounting to $121 for medications, $11,900 for hospitalizations, $118,510 for consultations, and $120 for clinical studies annually. Cost-effectiveness analyses of monotherapy and bitherapy treatments for Type 2 diabetes indicate that patient expenses, without public health subsidies, can range from $2468 to $15,500 depending on the treatment regimen [35]. The Mexican Institute of Social Security bears the highest costs associated with diabetes, largely due to the substantial demand for healthcare services [36]. Consequently, providing a natural and accessible therapeutic option that encourages a healthier lifestyle could greatly benefit both the population and the healthcare system.

Dietary recommendations: Healthcare providers may advocate for the inclusion of flaxseed in the dietary regimens of patients with obesity, prediabetes, or Type 2 diabetes due to its potential benefits on glucose and lipid metabolism [3,4,5,14,22,24,26,27,28,29]. Flaxseed supplementation has been associated with significant reductions in waist circumference and body composition among overweight and obese individuals, thereby supporting weight management initiatives [1,16,19,21,25,33]. Furthermore, flaxseed is recognized for its ability to lower LDL cholesterol levels and mitigate inflammation, which can enhance cardiovascular health in susceptible populations [12,15,17,23,30,31,32]. This dietary intervention may serve as a complementary strategy alongside conventional treatments for diabetes and related metabolic disorders, potentially decreasing the reliance on pharmacological therapies.

Lifestyle medicine perspective: Effective management of Type 2 diabetes requires a comprehensive, multidisciplinary approach centered on the patient (“Case Management”), from prevention to treatment and research [37,38]. This team approach aims to help the patient achieve prevention and general clinical well-being [39]. In this team—comprising patients, caregivers, physicians, endocrinologists, nutritionists, and other healthcare professionals—nurses play a vital role because they are closest to the patients [40]. Investing in the training and empowerment of these professionals to utilize lifestyle medicine represents a significant opportunity to improve chronic disease management. This approach can foster a more supportive community environment for integrated and modern care. Specifically, integrating lifestyle medicine skills into the skillsets of health professionals already in practice offers a practical and immediate solution to address pressing healthcare challenges, ultimately contributing to an improved quality of life for vulnerable populations affected by Type 2 diabetes and other chronic diseases [41].

Further research: Researchers are encouraged to conduct further clinical trials in diverse populations to fully explore the potential of flaxseed in managing and preventing Type 2 diabetes and its related complications.

### 4.2. Limits

Some limitations of the present study are that although several lifestyle factors were controlled for, these were outpatients who may or may not have reported any changes to the guidelines. Regarding flaxseed consumption, the principal investigator maintained constant communication with the participants to ensure that consumption was as directed.

Although participants were closely monitored to ensure compliance, self-reported consumption may not be entirely reliable. Future studies should incorporate additional adherence measures, such as daily consumption logs or biomarker validation. Despite these limitations, the study demonstrated that incorporating flaxseed into the diet is a practical and effective strategy to improve biochemical markers in patients with uncontrolled Type 2 diabetes.

## 5. Conclusions

Adjuvant treatment with metformin plus flaxseed was more effective in normalizing levels of glycated hemoglobin, glucose, total cholesterol, and triglycerides in patients with Type 2 diabetes in Oaxaca, Mexico.

Healthcare providers may recommend incorporating flaxseed into the diets of patients with obesity, prediabetes, or Type 2 diabetes to improve glucose and lipid metabolism and overall metabolic health.

## Figures and Tables

**Table 1:** Baseline characteristics of study participants (*n* = 166).

Variables	Control Group (*n* = 84)	Flax Intervention Group (*n* = 82)	*p* Value
Age (years)	54.5 ± 7.4 (Min 34/Max 66)	54.6 ± 8.8 (Min 25/Max 66)	0.976
Gender % (*n*)			
Female	50 (42)	50 (41)	0.562
Male	50 (42)	50 (41)	
Smokers (%)	11.9	14.6	0.386
Drug addicts (%)	0	0	-
History of HBP (%)	39.3	53.7	0.219
History of T2D (%)	46.4	75.6	0.118
GH (mg/dL)	10.1 ± 2.0 (Min 6.7/Max 13.8)	10.1 ± 1.9 (Min 6.6/Max 13.9)	0.674
Glucose (mg/dL)	157.3 ± 66.5 (Min 84/Max 344)	174.9 ± 71.0 (Min 80/Max 364)	0.102
Total Cholesterol (mg/dL)	222.2 ± 38.9 (Min 115/Max 297)	205.0 ± 35.1 (Min 120/Max 317)	0.056
Triglycerides (mg/dL)	235.5 ± 56.6 (Min 127/Max 417)	252.0 ± 78.0 (Min 116/Max 551.1)	0.125

Min, minimum; Max, maximum; GH, glycated hemoglobin; HBP, high blood pressure; and T2D, Type 2 diabetes.

## Data Availability

The original contributions presented in this study are included in the article. Further inquiries can be directed to the corresponding author.
