# Correction: López-Toledo et al. Flaxseed Improves Glucose and Lipid Metabolism in Mexican Subjects with Type 2 Diabetes: A Parallel Randomized Clinical Trial. *Nutrients* 2025, *17*, 709

**DOI:** 10.3390/nu18010074

**Published:** 2025-12-26

**Authors:** Sabina López-Toledo, María Cruz Pineda De la Cruz, Itzae Adonai Gutiérrez-Hurtado, Ana L. Gijón-Soriano, Enrique Martínez-Martínez, Carlos Valencia-Santiago, José E. Orellana-Centeno, Sergio A. Ramírez-García, Royer Pacheco-Cruz

**Affiliations:** 1 Center for Studies in Health Sciences and Disease, “Benito Juarez” Autonomous University of Oaxaca, Oaxaca 68000, Mexico; 2 “Presidente Benito Juárez” Hospital, Oaxaca 68040, Mexico; 3 Department of Molecular Biology and Genomics, University Center for Health Sciences, University of Guadalajara, Guadalajara 44340, Mexico; 4 Faculty of Dentistry, “Benito Juarez” Autonomous University of Oaxaca, Oaxaca 68120, Mexico; 5 Faculty of Medicine, “Benito Juarez” Autonomous University of Oaxaca, Oaxaca 68000, Mexico; 6 Faculty of Chemical Sciences, “Benito Juarez” Autonomous University of Oaxaca, Oaxaca 68000, Mexico; 7 Health and Civil Protection Directorate of Ejutla de Crespo, Oaxaca 71500, Mexico

**Error in Figure**

In the original publication [1], there was a mistake in Figure 3 as published. The same data were inadvertently duplicated from Figure 2, resulting in a misrepresentation of the control group data. The corrected Figure 3 appears below.

The authors state that the scientific conclusions are unaffected. This correction was approved by the Academic Editor. The original publication has also been updated.

## Figures and Tables

**Figure 3:**
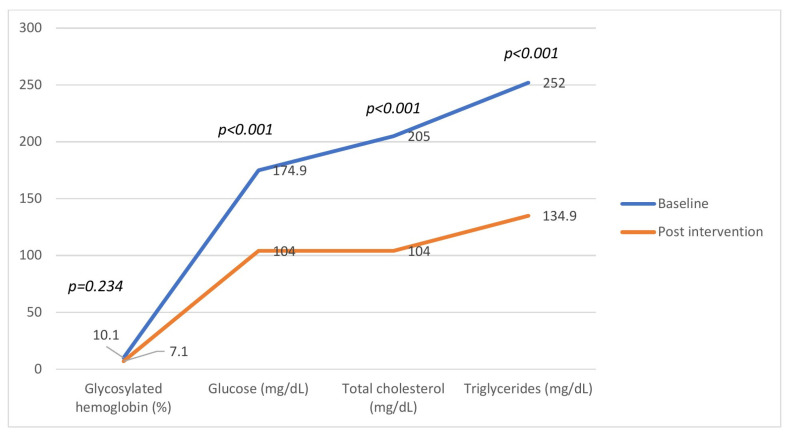
Behavior of biochemical parameters of participants in the intervention group (*n* = 82) before and after 12 weeks of flaxseed supplementation. Significant reductions were observed in fasting glucose, total cholesterol, and triglycerides (*p* < 0.001), while the change in glycated hemoglobin was not significant (*p* = 0.234). Data are presented as mean ± SD.
